# The association between exposome score for schizophrenia and metabolic parameters in individuals with schizophrenia and healthy controls: Findings from the EUGEI study

**DOI:** 10.1192/j.eurpsy.2023.601

**Published:** 2023-07-19

**Authors:** G. Erzin, C. Atbaşoğlu, L. Fusar-Poli, M. C. Saka, A. Üçok, K. Alptekin, J. V. Os, G. Gümüş Akay, S. Gülöksüz

**Affiliations:** ^1^ Ankara Diskapi Training and Research Hospital; ^2^Ankara University, Medical Faculty, Psychiatry Department, ankara, Türkiye; ^3^University of Catania, Medical Faculty, Psychiatry Department, Catania, Italy; ^4^İstanbul University, Medical Faculty, Psychiatry Department, İstanbul; ^5^Dokuzeylül University, Medical Faculty, Psychiatry Department, İzmir, Türkiye; ^6^Utrecht University, Medical Center, Psychiatry Department, Utrecht, Netherlands; ^7^Ankara University, Medical Faculty, Physiology Department, Ankara, Türkiye; ^8^Maastricht University, Psychiatry and Neuropsychology Department, Maastricht, Netherlands

## Abstract

**Introduction:**

Exposome is all nongenetic exposures from the prenatal period to death. Exposome score for schizophrenia (ES-SCZ) is a cumulative measure of environmental liability for schizophrenia. Our previous studies showed that the ES-SCZ is associated with mental and physical health outcome.

**Objectives:**

The aim of the study is to investigate the association of the ES-SCZ with metabolic parameters in individuals with schizophrenia and healthy controls.

**Methods:**

This study obtained 124 individuals with schizophrenia and 440 healthy controls from the European Network of National Schizophrenia Networks Studying Gene-Environment Interactions, Work Package 6 (Vulnerability and Severity) Turkey dataset. The ES-SCZ was calculated by summing log-odds weighted environmental exposures (childhood adversities, winter birth, hearing impairment and cannabis use). Linear regression analysis was used to investigate the association between ES-SCZ and metabolic parameters. After that analysis age and sex were added as covariates.

**Results:**

There was an association between ES-SCZ and diastolic blood pressure (B = -2.69 [95% CI -4.74; -.65], P-value = 0.010) in schizophrenia. ES-SCZ was associated with the fasting glucose level (B = -6.23 [95% CI -11.59; -.87], P-value = 0.023); high density lipid level (B = 1.77 [95% CI .27; 3.27], P-value = 0.021) in control and these results remained significant after adjusting for age and sex.

**Conclusions:**

ES-SCZ was associated with important metabolic parameters. These findings show that ES-SCZ is not only related to increasing the risk for psychosis development but may also influence comorbidities. This result is important since it may increase our knowledge of ES-SCZ and contribute to the importance and framework of its clinical implementation.

**Disclosure of Interest:**

None Declared

